# *Toxoplasma gondii* sustains survival by regulating cholesterol biosynthesis and uptake via SREBP2 activation

**DOI:** 10.1016/j.jlr.2024.100684

**Published:** 2024-10-28

**Authors:** Yi-Min Fan, Qing-Qi Zhang, Ming Pan, Zhao-Feng Hou, Lizhi Fu, Xiulong Xu, Si-Yang Huang

**Affiliations:** 1 Institute of Comparative Medicine, College of Veterinary Medicine, Yangzhou University, and Jiangsu Co-innovation Center for Prevention and Control of Important Animal Infectious Diseases and Zoonosis, and Jiangsu Key Laboratory of Zoonosis, Yangzhou, Jiangsu Province, PR China; 2 Joint International Research Laboratory of Agriculture and Agri-Product Safety, the Ministry of Education of China, Yangzhou University, Yangzhou, PR China; 3 Chongqing Academy of Animal Sciences, Chongqing, PR China

**Keywords:** SREBP2, *Toxoplasma gondii*, cholesterol, HMGCR, SQLE, LDLR

## Abstract

*Toxoplasma gondii* (*T. gondii*) is an obligate intracellular parasite that cannot biosynthesize cholesterol via the mevalonate pathway, it sources this lipid from its host. We discovered that *T. gondii* infection upregulated the expression of host cholesterol synthesis-related genes HMG-CoA reductase(HMGCR), squalene epoxidase (SQLE), and dehydrocholesterol reductase-7 (DHCR7), and increased the uptake pathway gene low-density lipoprotein receptor (LDLR). We found a protein, sterol regulatory element binding protein 2 (SREBP2), which is the key protein regulating the host cholesterol synthesis and uptake during *T. gondii* infection. *T. gondii* induced a dose-dependent nuclear translocation of SREBP2. Knockdown SREBP2 reduced *T. gondii*-induced cholesterol biosynthesis and uptake. Consequently, the parasite's ability to acquire cholesterol was significantly diminished, impairing its invasion, replication, and bradyzoites development. Interfering cholesterol metabolism using AY9944 effectively inhibited *T. gondii* replication. In summary, SREBP2 played an important role in *T. gondii* infection in vitro, serving as a potential target for regulating *T. gondii*-induced cholesterol metabolism, offering insights into the prevention and treatment of toxoplasmosis.

## Highlights

• *T. gondii* infection increased the expression of the cholesterol uptake gene *LDLR* and the biosynthesis genes *HMGCR*, *SQLE* and *DHCR7*.
• *T. gondii* infection increased the nuclear translocation of SREBP2.
• AY9944 reduced *T. gondii* replication.
• Activated SREBP2 regulated both the cholesterol uptake gene *LDLR*, and the biosynthesis genes *HMGCR*, *SQLE* and *DHCR7* during *T. gondii* infection.
• Limiting SREBP2 expression reduced cholesterol uptake and biosynthesis and limited T. gondii invasion, replication, and bradyzoites development.

## Graphical abstract

**Figure:**
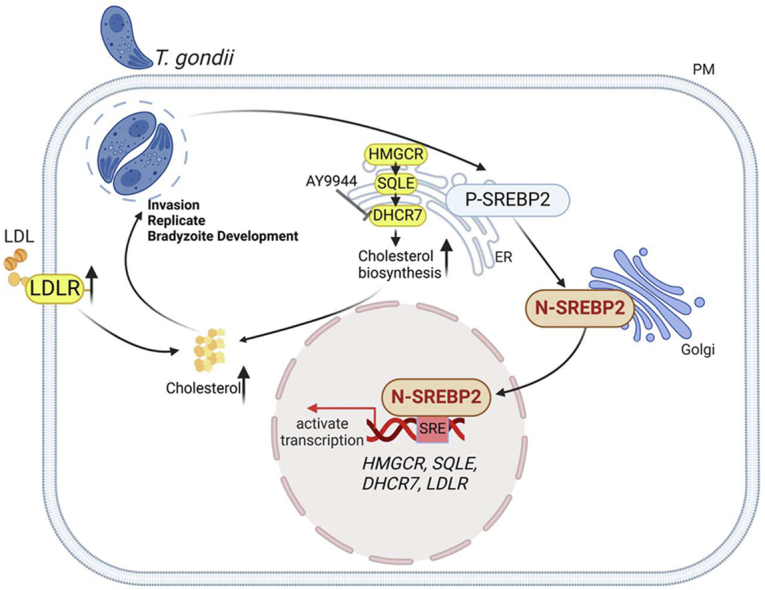

*Toxoplasma gondii* is an obligate intracellular parasite that infects all warm-blooded animals, including humans (1). In individuals with normal immune function, the infection is often asymptomatic, with mild symptoms that usually resolve within a few days to a few weeks. However, the parasite can persist in the host's brain, heart, and skeletal muscles in the form of cysts (2). In contrast, *T. gondii* infection poses severe risks to immunocompromised or immunodeficient individuals, such as pregnant women, patients with AIDs, and organ transplant recipients. It can lead to serious conditions including toxoplasmic encephalitis, chorioretinitis, necrotizing interstitial pneumonia, miscarriage, and stillbirth, and is a major cause of mortality in these patients (3). Currently, the treatment regimen for toxoplasmosis, comprising sulfadiazine and pyrimethamine, is only effective against the tachyzoites stage of acute infection, with significant side effects and a high rate of relapse (4). Additionally, there are no available drugs that can eliminate cysts, and chronic infection remains untreatable. This underscores the urgent need for novel therapeutic approaches.

*T. gondii* cannot biosynthesize cholesterol via the mevalonate pathway (5, 6, 7); it relies on salvaging and storing cholesterol from the host (8). The invasion relies on the formation of moving junctions, a process that requires host plasma membrane cholesterol (9). Shortly after the invasion, *T. gondii* rapidly incorporates cholesterol into the parasitophorous vacuoles (PVs). Previous studies have found cholesterol accumulation on the *T. gondii* plasma membrane and rhoptries (6, 9). When cellular cholesterol is depleted or lysosomal cholesterol transport is blocked by pharmacological agents, *T. gondii* exhibits abnormal budding and reduced replication. Due to host constraints, tachyzoites differentiates to bradyzoites, forming cyst walls to resist host clearance, bradyzoites divide slowly within the cyst wall (10). While the role of cholesterol related to the differentiation process remains unknown.

*T. gondii* infection alters host cell metabolism, including cholesterol uptake and biosynthesis. Uptake relied on the key gene low-density lipoprotein receptor (LDLR), and biosynthesis was regulated by several important genes including HMG-CoA reductase(HMGCR), squalene epoxidase (SQLE) and dehydrocholesterol reductase 7 (DHCR7) (11, 12). Several studies indicated that these genes could be exploited by viruses to enhance their invasion and infection processes (13, 14, 15). Intracellular cholesterol metabolism is tightly regulated by sterol regulatory-element binding protein 2 (SREPB2) which is a key transcription factor in cholesterol metabolism. SREBP2 can be activated through proteolytic cleavage, enabling it to translocate to the nucleus where it binds to sterol regulatory elements (SREs) in target genes, thereby promoting the expression of genes involved in cholesterol biosynthesis and uptake (16).

However, there is no information about how *T. gondii* regulates the host cholesterol uptake and biosynthesis. In this study, we aimed to investigate whether *T. gondii* infection activated host cholesterol biosynthesis and uptake, and the key genes involved in cholesterol uptake and biosynthesis were studied in the detailed mechanism of maintaining cholesterol homeostasis, during *T. gondii* invasion, replication, and bradyzoites development.

## Materials and methods

### Antibodies

The following antibodies were used in this study: anti-SREBP2 (R&D Systems, MAB7119), anti-LDLR (Invitrogen, MA5-23916), anti-HMGCR (sc-271595, Santa Cruz), anti-SQLE (sc-271651, Santa Cruz), anti-FDFT1 (sc-271602, Santa Cruz), anti-HMGCS1 (sc-373681,Santa Cruz), anti-MVD (sc-376975,Santa Cruz), anti-TgSAG1 (MA5-18268, Invitrogen), anti-TgIMC1 (a kind gift), anti- GAPDH (G8795, Sigma-Aldrich), Anti-T. gondii Polyclonal Antibody(PA1-7252, Invitrogen), Dolichos Biflorus Agglutinin (DBA, FL-1031,Vector).

### Reagents

AY9944 (A8658) was purchased from APExBIO. Pyrimethamine (HY-18062) was purchased from MedChemExpress. Water-soluble cholesterol (C4951) was purchased from Sigma-Aldrich. Filipin III (480-49-9) was purchased from Cayman Chemical. TRIzol (15596026), fluorescent secondary antibodies, and Lipofectamine 2000 were purchased from Invitrogen. cDNA Synthesis Kit (R212-02), SYBR qPCR Master Mix(Q312-02), and Annexin V-FITC/PI apoptosis detection kit (A211) were purchased from Vazyme. A genomic DNA kit (DP304) was purchased from TIANGEN. A lactate dehydrogenase (LDH) release assay kit was purchased from Beyotime. Protease and phosphatase inhibitor cocktail (P002) and Chemiluminescence assay kit (P10300,P10060) were purchased from NCM Biotech. Dulbecco‘s modified Eagle’s medium(DMEM), OPTI-MEM, and 1640 were purchased from Gibco. FBS was purchased from EallBio.

### Mammalian cell and parasite culture

Human cervical carcinoma (HeLa) and human foreskin fibroblast (HFF) cells were maintained in DMEM supplemented with 10% FBS, L-glutamine, and penicillin-streptomycin at 37°C and 5% CO2. NL20 cells were grown in Ham's F12 medium with 4% FBS. RH, RFP-RHm and PRU strains of *T. gondii* were cultured on HFF cells in DMEM with 1% FBS.

### Western blotting analysis

Samples were collected and lysed in an SDS Sample Buffer and separated by SDS-PAGE, transferred to polyvinylidene fluoride membranes. Membranes were blocked with 5% BSA in TBST then probed with primary antibodies for SREBP2(1:4000), LDLR(1:1000), HMGCR(1:500), SQLE(1:1000), FDFT1(1:1000), HMGCS1(1:1000), MVD(1:1000), TgSAG1(1:2000), or GAPDH overnight at 4°C. Membranes were developed using a chemiluminescence assay kit and analyzed by ImageJ software. To isolate nuclear proteins for N-terminal of SREBP2 (N-SREBP2) detection, cells were washed twice with ice-cold PBS and lysed in 200 μl of hypotonic buffer (20 mM Tris-HCl, pH 7.4, 10 mM NaCl, 3 mM MgCl2) containing protease and phosphatase inhibitor cocktail for 15 min. Subsequently, 10 μl of 10% NP-40 was added, and the mixture was vortexed at maximum speed for 10 s. The sample was then centrifuged at 3000 rpm for 10 min. The resulting pellet was lysed in NP-40 buffer (50 mM Tris (pH 8.0), 150 mM NaCl, 5 mM EDTA, 1% NP-40) with protease and phosphatase inhibitor cocktail for 15 min. After ultrasonication were immediately heated at 100°C.

### Real-time quantitative PCR analysis

Cells grown in 12-well plates were harvested 24 h post-infection (hpi). Total RNA was isolated using a Trizol reagent according to the manufacturer’s instructions. Reverse transcription of purified RNA was converted to cDNA, and quantified by real-time PCR(qPCR) with SYBR Green. All primers used in this study were as follows *SREBF2*-F: AACGGTCATTCACCCAGGTC; *SREBF2*-R: GGCTGAAGAATAGGAGTTGCC; *HMGCR*-F: CCCAGCCTACAAGTTGGAAA; *HMGCR*-R: GCTCCCATCACCAAGGAGTA; *SQLE*-F:GGCCATCTTTTGTTGGAGAA; *SQLE*-R: TTCAGAAGGGAATGGGAGTG; *DHCR7*-F: CCAGAATTCTATGGCTGCAAAATCGCAAC; *DHCR7*-R: ATTCTCGAGGAAGATTCCAGGCAGCAG.

### Quantification of parasites

The anti-*T. gondii* activity of AY9944 was evaluated by an intracellular growth assay. AY9944 was diluted to a 10 mM solution in water to prepare a stock solution. Pyrimethamine (PYR) was diluted to 5 mM solution in DMSO. For stimulation, HeLa cells were primed with AY9944 or PYR for 4 h. The freshly released tachyzoites of the RH-RFP strain were added to cells in 96-well plates at 2 hpi, and the infection medium was replaced with 1640 1% FBS containing either AY9944 (5 μM), vehicle control, or PYR (10 μM, positive control) in each well. After 24 hpi, the growth of RH-RFP was observed and photographed under a fluorescence microscope. The absorbance was then measured at 532 nm using a microplate reader. The same treatment was applied, and DNA was collected after 24 hpi for future analysis.

A PCR-based assay was used to determine the number of parasites in host cells as previously described (17). Briefly, HeLa cells were maintained in 1640 5% FBS. The cells were infected with freshly released RH at 2 hpi, and the infection medium was replaced with 1640 1% FBS. At 24 hpi, genomic DNA was isolated and measured by qPCR using primers to a parasite-specific gene region designated *B1*. The primers were as follows: Tg*B1-*F:AACGGGCGAGTAGCACCTGAG; Tg*B1-*R:TGGGTCTACGTCGATGGCATGACAAC. Parasite viability/replication was determined by counting the number of parasites per vacuole in more than 100 vacuoles.

### Immunofluorescence assay

The RH strains were inoculated into HeLa cells on glass coverslips. After 24 h of infection, the cells were fixed and permeabilized. Subsequently, the cells were stained with mouse monoclonal anti-SREBP2 (1:200) and rabbit polyclonal anti-TgIMC1 at 4°C overnight. Alexa Fluor™ 488-conjugated (green) goat anti-mouse IgG, Alexa Fluor™ 594-conjugated (red) goat anti-rabbit IgG, and DAPI dye were used for antigen and DNA visualization.

Filipin staining was performed as described previously (18). Briefly, after fixation, cells were stained with filipin III (50 μg/ml) for 2 h at room temperature. SYTOX Green was used for DNA visualization. To measure cellular cholesterol levels, WT and KO cells were harvested and then fixed with 4% paraformaldehyde for 10 min, then centrifuged at 2000 rpm for 5 min. Filipin staining was performed for 1 h, after which the cells were washed once with PBS and resuspended in 400 μl of PBS. Data were acquired using a flow cytometer (FACS LSRFortessa, BD Biosciences), and fluorescence intensity was analyzed using FlowJo software.

A green/red invasion assay was performed as described previously (19). In brief, freshly egressed tachyzoites were introduced into HeLa cells seeded on glass coverslips. After a 30-minute and 60-minute invasion period, an immunofluorescence assay was carried out to differentiate between invaded and non-invaded parasites. Extracellular parasites were labeled with rabbit anti-*T. gondii* polyclonal antibody before cell permeabilization and then stained with Alexa Fluor™ 594-conjugated (red) goat anti-rabbit IgG. All parasites were subsequently labeled with mouse anti-TgSAG1 antibodies and stained with Alexa Fluor™ 488-conjugated (green) goat anti-mouse IgG. At least 100 parasites were counted for each sample.

### Bradyzoites differentiation and replication assay

The Pru strains were inoculated into WT and SREBP2 knockdown cells on glass coverslips. The invaded tachyzoites were maintained into bradyzoites using normal DMEM or alkaline 1640 medium with 2% FBS at 2 hpi. For alkaline induction, the cultures were kept in a CO₂-free incubator at 37°C for 7 days, with the medium replaced every 48 h to sustain the alkaline conditions. The conversion of tachyzoites to bradyzoites and the intracellular replication capacity of bradyzoites were assessed using an immunofluorescence assay. Tachyzoites and the cyst wall were stained with mouse anti-TgSAG1 antibody and Dolichos Biflorus Agglutinin (DBA), and fluorescein (green), respectively. TgSAG1-positive, DBA-positive, and double-positive PVs in each group were selected for statistical analysis. The number and relative size of bradyzoites were quantified using Image J.

### Cell viability

The HeLa cells were treated for 24 h in combination with AY9944(10 μM). The cell viability was measured using the Annexin V-FITC/PI Apoptosis Detection Kit according to the manufacturer’s protocol. The HeLa cells were plated in a 96-well plate with 10% FBS DMEM, then treated for 4 h with AY9944(5 μM) and PYR(10 μM). The freshly released tachyzoites of the RH-RFP strain were added to cells in the 96-well plates, the infection medium was replaced with 1640 1% FBS containing either AY9944 (5 μM), vehicle control, or PYR (10 μM, positive control) was added to each well at 2 hpi. After 36 hpi, viability was measured using the LDH Cytotoxicity Assay according to the manufacturer’s protocol.

### SREBP2 knockdown cell line

HeLa cells were maintained in DMEM with 10% FBS in 24-well plates. The shRNA targeting human SREBP-2 (sc-36559) and shRNA control (sc-37007) were purchased from Santa Cruz Biotechnology. The cells were transfected with shRNA for 24 h using Lipofectamine 2000, according to the manufacturer’s instructions. Stable shRNA transfections were selected in a medium containing 0.8 μg/ml puromycin. Drug-resistant colonies were pooled and analyzed for SREBP2 expression by Western blot.

### Quantification and statistical analysis

The data used in the study have been expressed as mean ± SD. Statistical differences between the experimental groups were calculated using Graph Pad Prism 8.1 software. A value of *P* < 0.05 was considered statistically significant. All experiments were performed at least three times.

## Results

### *T. gondii* infection increased the expression of genes regulating cholesterol biosynthesis and uptake

**Fig. 1:**
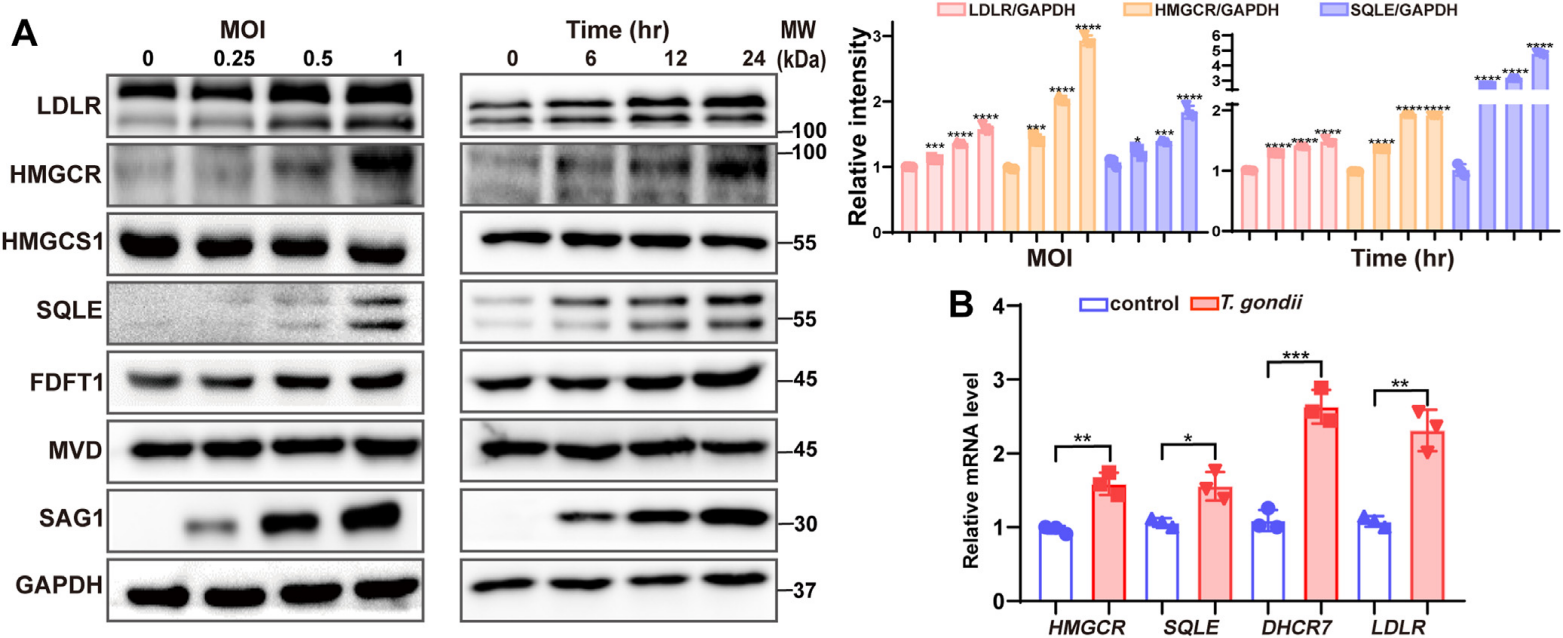
*T. gondii* infection upregulates LDLR, HMGCR, SQLE, and DHCR7 expression. A: HeLa cells were infected with the indicated multiplicity of infection (MOI) of RH (0, 0.25, 0.5, and 1) for 24 h, or with an MOI of 0.5 for varying durations. Levels of LDLR, HMGCR, HMGCS1, SQLE, FDFT1, MVD, and *T. gondii* SAG1 proteins were analyzed by Western blot. GAPDH served as a loading control. The density of the bands was analyzed using Image-J software and normalized to the arbitrary units of GAPDH. B: The mRNA levels were quantified using qPCR at 24 hpi. Statistical significance was denoted as ∗*P* < 0.05, ∗∗*P* < 0.01, ∗∗∗*P* < 0.001, ∗∗∗∗*P* < 0.0001 compared to uninfected controls.

**Supplemental Figure S1:**
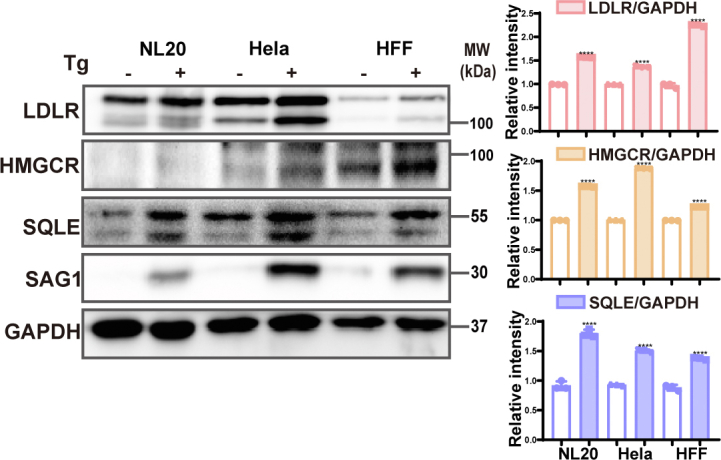
*T. gondii* infection induced LDLR, HMGCR, and SQLE activation, related to Figure 1. HeLa, NL20, and HFF cells were infected with RH for 24 h. The levels of LDLR, HMGCR, SQLE, and *T. gondii* SAG1 proteins were analyzed by western blot, with GAPDH detected as a loading control.

Previous studies indicated that *T. gondii* infection altered cellular cholesterol metabolism by microarray hybridization (20). To further understand the regulation of host cholesterol biosynthesis and uptake, qPCR and Western blot were used to evaluate the expression of genes related to biosynthesis, including cholesterol biosynthesis genes HMGCR, HMG-CoA synthase (HMGCS1), SQLE, farnesyl-diphosphate farnesyltransferase 1 (FDFT1), mevalonate diphosphate decarboxylase (MVD), and uptake gene LDLR in *T. gondii* infected HeLa cells. Western Blot analysis showed that the expression of HMGCR, SQLE, and LDLR increased in a manner of time- and dose-dependent in HeLa cells during *T. gondii* infection (Fig. 1A). The same results were also found in NL20 and HFF cells (supplemental Fig S1). Furthermore, qPCR was used to confirm the above results (Fig. 1B). Recently, research has shed light on the significant role of DHCR7 in the replication of various viruses (15). Here, we demonstrated that *T. gondii* upregulated the expression of DHCR7. Overall, these results showed that *T. gondii* infection induced the expression of HMGCR, SQLE, DHCR7, and LDLR in HeLa cells.

### The DHCR7 inhibitor AY9944 reduced *T. gondii* replication in HeLa cells

**Fig. 2:**
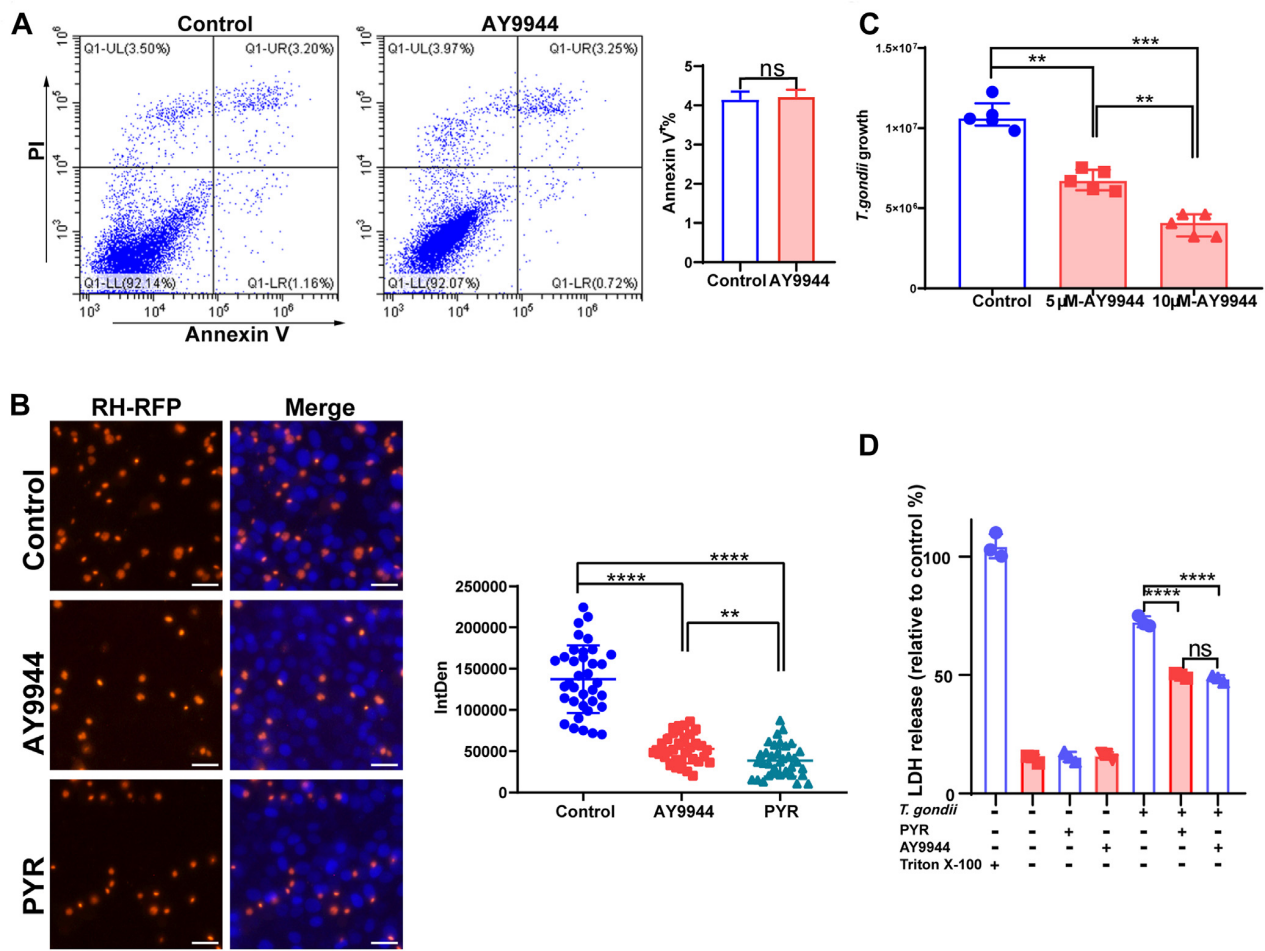
The DHCR7 inhibitor AY9944 reduced *T. gondii* replication. A: HeLa cells were treated with AY9944 (10 μM) for 24 h to analyze cell apoptosis using PI and Annexin V staining. B: RH-RFP were cultured in the presence of AY9944 (5 μM) or PYR (10 μM) in 96-well plates for 24 h. The absorbance was measured at 532 nm using a microplate reader. C: RH were cultured in the presence of different doses of AY9944 in HeLa cells for 24 h, and then parasite load was quantified by qPCR. Images were taken at 200x magnification. Scale bar: 20 μm. D: LDH release expressed as a percentage of the positive control (lysed cells) was determined after incubating HeLa cells with drugs at 36 hpi. ∗*P* < 0.05, ∗∗*P* < 0.01, ∗∗∗*P* < 0.001, ∗∗∗∗*P* < 0.0001, compared to controls.

*T. gondii* relies on scavenging cholesterol from the host for its survival. We demonstrated that the expression of DHCR7, the terminal enzyme of cholesterol biosynthesis, was upregulated following infection. We hypothesized that reduced DHCR7 activity will decrease the *T. gondii* replication. Pharmacological inhibition AY9944 was used to inhibit DHCR7 activity in HeLa cells, from the results we found that AY9944 did not affect cell viability at a concentration of 10 μM (Fig. 2A). Cells were pre-treated with 5 μM AY9944 for 4 h to deplete cellular cholesterol, freshly egressed *T. gondii* was then added to the cells for 2 h, non-invading parasites were washed away, and the medium was replaced with fresh medium containing 5 μM AY9944. The cells were incubated for an additional 24 h, then the fluorescence intensity of RH-RFP was measured, pyrimethamine (PYR) was used as a control. Our results showed that both AY9944 and PYR significantly inhibited the intracellular proliferation of *T. gondii* (Fig. 2B). To determine the specificity of this inhibitory effect, cells were treated with varying doses of AY9944 and *T. gondii* genes was evaluated by qPCR (Fig. 2C). The effect of drug treatment on cell viability in *T. gondii* infection was assessed using the LDH assay. The *T. gondii* infection increased LDH release from cells, while treatment with AY9944 or PYR reduced LDH release in infected cells (Fig. 2D). The results demonstrated that the inhibition of *T. gondii* replication showed a dose-dependent manner. Together, these results indicated that inhibiting DHCR7 activity results in replication defects in *T. gondii*.

### *T. gondii* infection increased the SREBP2 which regulates cholesterol homeostasis

**Fig. 3:**
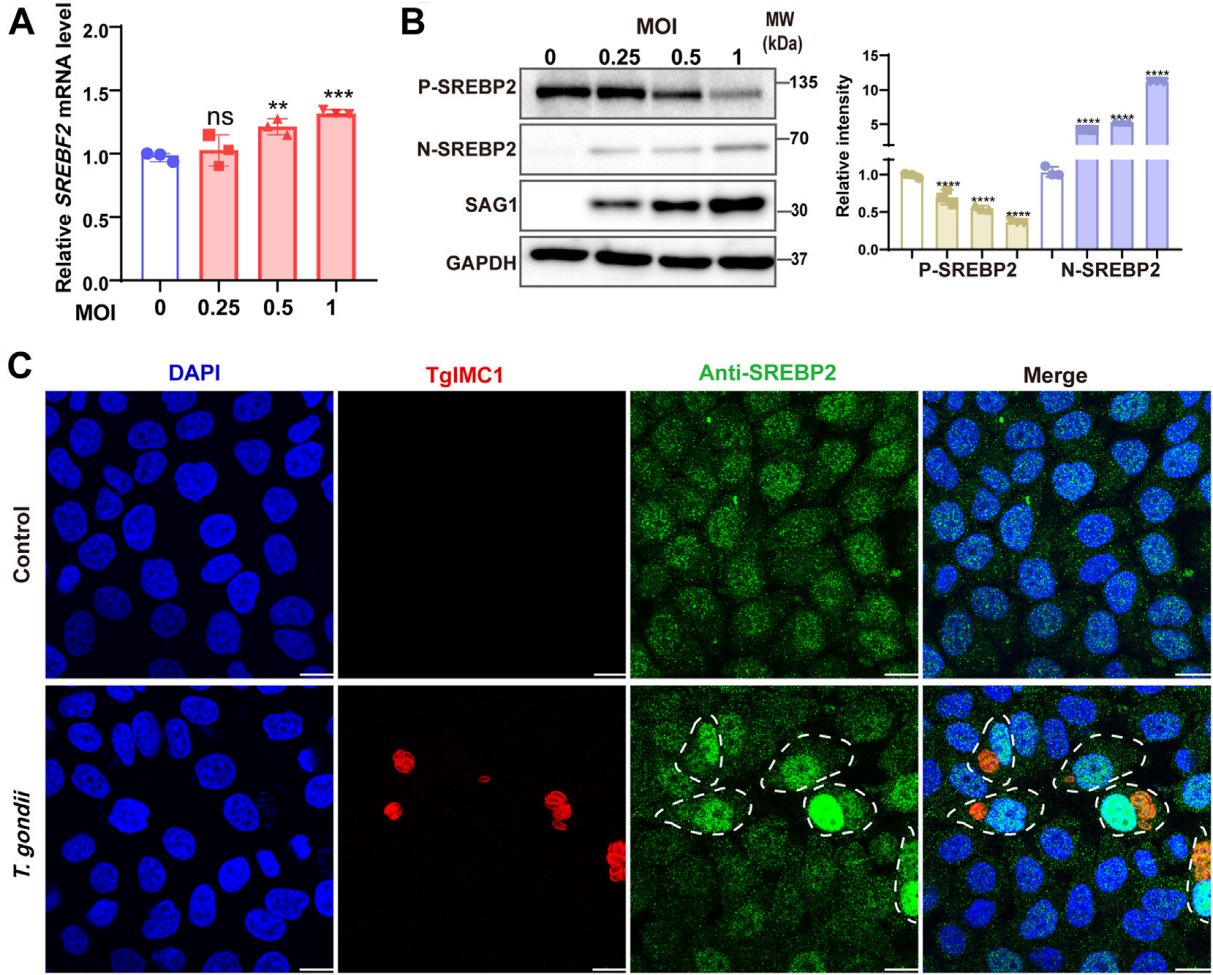
*T. gondii* infection induced SREBP2 activation. HeLa cells were infected with the indicated MOI (0, 0.25, 0.5, and 1) of RH for 24 h. A: the relative expression of *SREBF2* mRNA was measured by qPCR. B: the levels of SREBP2 protein were analyzed by Western blot. The density of the bands was analyzed using Image-J software and normalized to the arbitrary units of GAPDH. C: the cells were immunostained for anti-SREBP2 (*green*), anti-TgIMC1 (*red*) and the nuclei were stained using DAPI (*blue*), with images taken at 1000x magnification. *Scale bar*: 10 μm. ∗*P* < 0.05, ∗∗*P* < 0.01, ∗∗∗*P* < 0.001, ∗∗∗∗*P* < 0.0001, compared to controls.

**Supplemental Figure S2:**
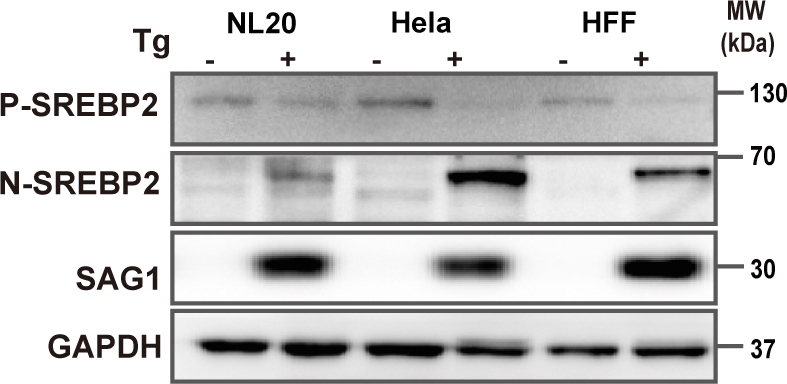
*T. gondii* infection induced SREBP2 activation, related to Figure 3. HeLa, NL20, and HFF cells were infected with RH for 24 h. The levels of SREBP2 were analyzed by western blot.

Cells acquire cholesterol through two pathways: biosynthesis and uptake. The aforementioned experiments demonstrated that *T. gondii* infection activates both pathways. Moreover, interfering with the final step of cholesterol biosynthesis via DHCR7 affected the survival of *T. gondii*. We aimed to identify key proteins that regulated these two pathways. Previous studies indicated that SREBP2 played important roles in both cholesterol biosynthesis and uptake. The role of SREBP2 remains poorly understood in *T. gondii* infection. Here, we found an increased expression of SREBP2 during *T. gondii* infection (Fig. 3A), and Western blot analysis showed that the level of the active form of N-terminal of SREBP2 (N-SREBP2) was increased after infection (Fig. 3B). The same results were also found in NL20 and HFF cells (supplemental Fig S2). The N-SREBP2 enters the nucleus, thereby driving the increased transcription of relevant target genes. We observed a significant increase of N-SREBP2 in the nuclear when the host cells were infected with *T. gondii* (Fig. 3C). These results indicated that host SREBP2 could be activated by *T. gondii*.

### Knockdown or inhibition SREBP2 decreased cholesterol biosynthesis and uptake which reduced *T. gondii* replication

**Fig. 4:**
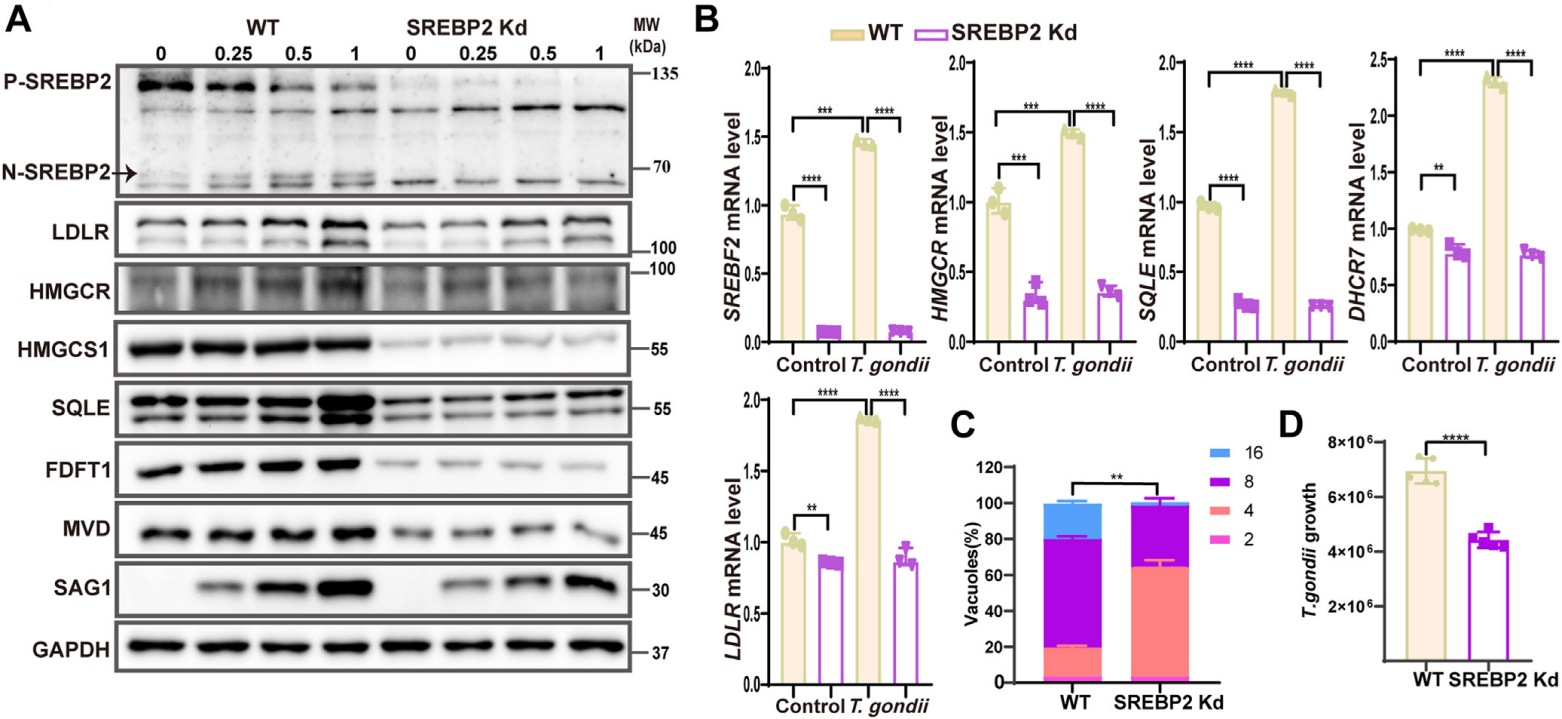
Knockdown SREBP2 reduced *T. gondii* infection by decreasing cholesterol biosynthesis and uptake. A: HeLa cells were infected with the indicated MOI (0, 0.25, 0.5, and 1) of RH for 24 h. Levels of SREBP2, LDLR, HMGCR, HMGCS1, SQLE, FDFT1, MVD, and *T. gondii* SAG1 proteins were analyzed by Western blot. GAPDH was used as a loading control. The N-SREBP2 band was indicated with an arrow. B: HeLa cells were infected with RH for 24 h. The relative expression of *SREBF2*, *HMGCR*, *DHCR7*, *SQLE*, and *LDLR* mRNA was measured by qPCR. C: HeLa cells were infected with RH for 24 h. Tachyzoites per PV were counted in 100 PVs. D: parasite load was quantified by qPCR. ∗*P* < 0.05, ∗∗*P* < 0.01, ∗∗∗*P* < 0.001, ∗∗∗∗*P* < 0.0001, compared to controls.

We hypothesized that inhibiting SREBP2 activity could reduce cellular cholesterol levels and would potentially affect the survival of *T. gondii*. To test this hypothesis, SREBP2 was knocked down in HeLa cells (Fig. 4A, B). TgSAG1 expression level was used to detect T. gondii replication, and the Western blot analysis results revealed that T. gondii replication was significantly reduced in SREBP2 knockdown cells (Fig. 4A). The same results were confirmed by calculating the number of tachyzoites in the PVs and measuring T. gondii DNA copy number (Fig. 4C, D).

**Supplemental Figure S3:**
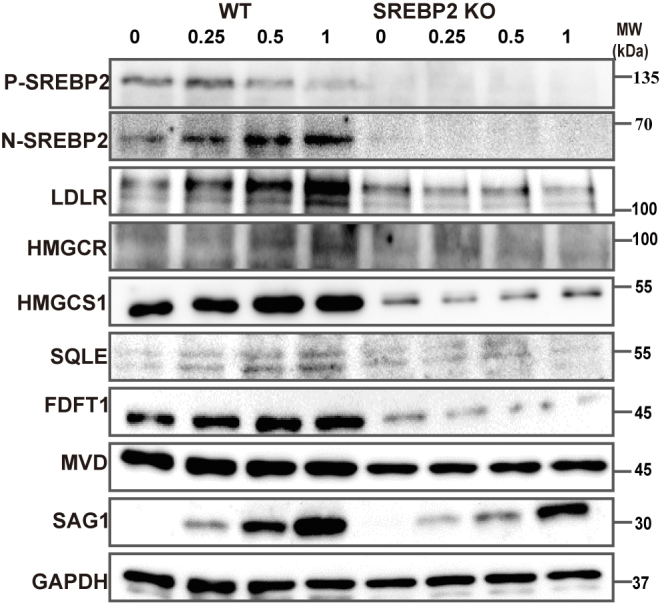
SREBP2 deficiency decreased the levels of LDLR, HMGCR, SQLE, and *T. gondii* proteins in NL20 cells, related to Figure 4. NL20 cells were infected with the indicated MOI (0, 0.25, 0.5, and 1) of RH for 24 h. The levels of SREBP2, LDLR, HMGCR, HMGCS1, SQLE, FDFT1, MVD, and the *T. gondii* SAG1 proteins were analyzed by western blot. GAPDH was detected as a loading control.

The aforementioned results demonstrated that *T. gondii* activated SREBP2, while inhibiting SREBP2 resulted in reduced *T. gondii* replication. We aimed to investigate the regulatory role of SREBP2 in cholesterol biosynthesis and uptake pathways during *T. gondii* infection. In SREBP2 knockdown cells, we found that the expression of LDLR, HMGCR, and SQLE decreased in all dose of *T. gondii* infection. Additionally, the levels of HMGCS1, FDFT1, and MVD were significantly downregulated in the knockdown cells (Fig. 4A). The same result was also found in SREBP2 knockdown NL20 cells (supplemental Fig S3). *T. gondii* could not upregulate the mRNA expression of *HMGCR*, *DHCR7*, *LDLR*, and *SQLE* in SREBP2 knockdown cells (Fig. 4B). Our results showed that these upregulated genes by *T. gondii* were significantly reduced in SREBP2 knockdown cells in a dose-dependent manner, indicating that *T. gondii* activated SREBP2 to modulate host biosynthesis and uptake. Overall, these results demonstrated that *T. gondii* infection induced host cholesterol biosynthesis and uptake by activating SREBP2.

### Knockdown SREBP2 reduced cholesterol accumulation on *T. gondii* and limited *T. gondii* invasion

**Fig. 5:**
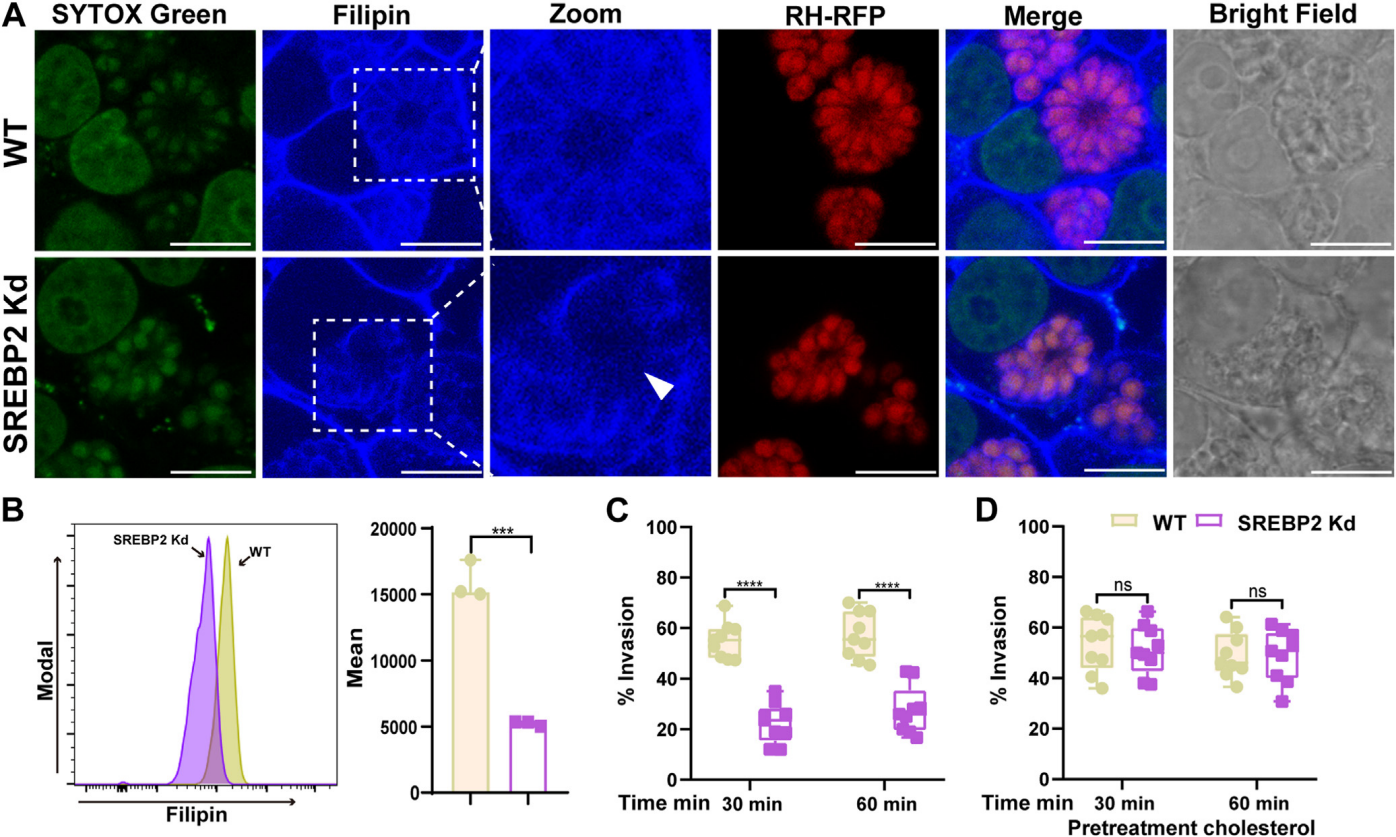
Knockdown SREBP2 reduced cholesterol accumulation on *T. gondii* and decreased *T. gondii* invasion in HeLa cells. A: HeLa cells were infected with RH-RFP for 36 h, fixed, and cytochemically stained with filipin for cholesterol detection. Cells were observed using confocal microscopy. The arrowhead indicates defects in tachyzoites in SREBP2 knockdown cells. RH-RFP (*red*), cholesterol stained with filipin (*blue*), and nucleus (*green*). B: the mean fluorescence intensity of Filipin staining was measured by flow cytometry. (C) invasion assay for HeLa cells infected with the RH were cultivated for 30 min or 60 min in normal medium (C) or in medium containing 2 μM cholesterol (D) ∗∗∗*P* < 0.001, ∗∗∗∗*P* < 0.0001.

The aforementioned results demonstrated that *T. gondii* upregulated the expression of genes involved in cholesterol biosynthesis and uptake through the activation of SREBP2. Therefore, the parasite might activate SREBP2 to obtain sufficient cholesterol for its survival. We used filipin, a specific fluorescent marker for unesterified cholesterol, to trace cholesterol. Our data showed that SREBP2 knockdown cells significantly reduced cholesterol accumulation on tachyzoites (Fig. 5A). The mean fluorescence intensity of Filipin staining was measured by flow cytometry, and the results showed that cholesterol levels were significantly reduced in SREBP2 knockdown cells(Fig. 5B). Our study demonstrated that *T. gondii* obtained sufficient host cholesterol via increased SREBP2.

To investigate the impact of SREBP2 on *T. gondii* biological properties, parasite invasion was examined. Using a green/red invasion method, we assessed *T. gondii* invasion under normal culture conditions and found that the invasion rate in SREBP2 knockdown cells was significantly reduced at 30 min and 60 min post-infection (Fig. 5C). To explore the relationship between reduced invasion and cholesterol, cholesterol was supplemented for 12 h and then *T. gondii* invasion was measured. Our results showed that the inhibitory effect of the invasion could be compensated for by supplementing with cholesterol in SREBP2 knockdown cells (Fig. 5D). These results indicated that reduced host cholesterol levels led to a decrease in *T. gondii* invasion.

### Knockdown SREBP2 reduced the number and size of *T. gondii* cysts

**Fig. 6:**
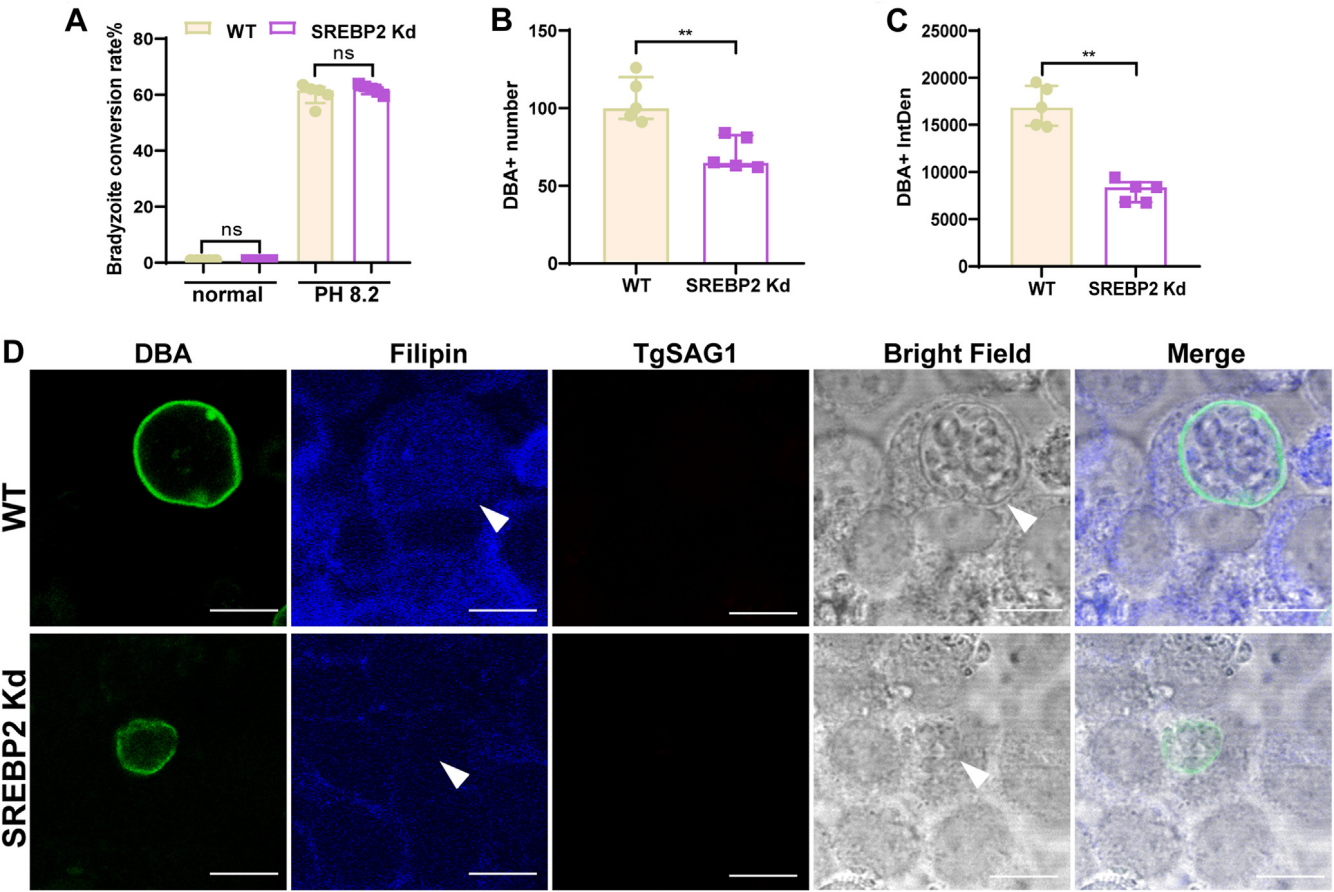
Knockdown SREBP2 reduced the number and size of *T. gondii* cysts in HeLa cells. Quantifications of day 7 in vitro cysts formed by the Pru strain under the indicated conditions: A: bradyzoite differentiation rate, calculated as DBA^+^PV/(DBA^+^ + SAG1^+^PV). B: number of cysts. C: relative size of cysts. TgSAG1 was labeled with Fluor 516 (*red*), and the cyst wall was labeled with biotinylated Dolichos biflorus agglutinin (DBA, *green*). D: IFA images of day 7 in vitro cysts formed by the Pru strain in a pH 8.2 medium containing 2% FBS. TgSAG1 was labeled with Fluor 516 (*red*), cholesterol with filipin (*blue*), and the cyst wall with DBA (*green*). Images were taken at 1000x magnification. Scale bar: 10 μm.

To avoid being clear by the host, tachyzoites can transform into bradyzoites and persist in tissues for long periods in the form of cysts (21). The conversion of tachyzoites into bradyzoites can be initiated in vitro by a variety of stresses, including alkaline pH, heat shock, nutrient starvation, or the use of specific drugs (22). Thus, we sought whether inhibited SREBP2 results in the development of tachyzoites to bradyzoites. Quantification showed that SREBP2 had no effect on the conversion of bradyzoites, no matter under normal conditions or alkaline pH stress (Fig. 6A). Furthermore, we quantified the number and relative size of cysts formed in SREBP2 knockdown cells under alkaline pH stress. The results showed that both the number and relative size of cysts were significantly reduced in SREBP2 knockdown cells (Fig. 6B–D). Collectively, these observations indicated that *T. gondii* activated the host's SREBP2-regulated cholesterol pathway which is crucial for bradyzoites growth and replication.

## Discussion

*T. gondii* cannot synthesize cholesterol via the mevalonate pathway, so it depends on the host's cholesterol (8, 23, 24). In our study, *T. gondii* infection upregulated cholesterol uptake and biosynthesis, which was significantly associated with changes in LDLR, HMGCR, SQLE, and DHCR7, respectively. Several studies indicated that reducing the activity of HMGCR or SQLE inhibited *T. gondii* replication (25, 26). DHCR7 was involved in the final step of cholesterol biosynthesis, and AY9944, an inhibitor of DHCR7, reduced host cholesterol levels (27). However, the role of DHCR7 in *T. gondii* replication is not available. Our data showed that inhibited DHCR7 with AY9944 significantly reduced *T. gondii* replication, which indicated DHCR7 had an important role in infection.

To maintain intracellular cholesterol levels, cholesterol biosynthesis and uptake were tightly regulated, SREBP2 is one of key regulatory factors (28), which was induced during viral infection, and its active form (N-SREBP2) was translocated to the nucleus (29, 30). Here, we found that nuclear translocation of SREBP2 increased in cells infected with *T. gondii*, and the pathway manipulation might serve multiple purposes. Our data suggested that the activation of SREBP2 might have benefited *T. gondii* survival.

After N-SREBP2 enters the nucleus, it binds to SREs, initiating cholesterol biosynthesis and uptake (31, 32). Similarly, we found that the levels of cholesterol biosynthesis-related proteins were significantly reduced in SREBP2 knockdown cells. Additionally, *T. gondii* upregulated the expression of LDLR, HMGCR, SQLE, and DHCR7 was also decreased in SREBP2 knockdown cells, which indicated that *T. gondii* upregulated these genes through SREBP2. SREBP2 is linked to immune pathways, where the host immune response produces 25-HC to inhibit infection by targeting SREBP2, thereby reducing available cholesterol. *T. gondii* may activate SREBP2 in an attempt to counteract this process (33). Previous researches indicated that SREBP2 activation increased host cholesterol, facilitating viral replication (30). In this study, we found that *T. gondii* replication was significantly impaired in SREBP2 knockdown cells, which suggested that *T. gondii* regulated cholesterol biosynthesis and uptake by activating SREBP2, which in turn promotes *T. gondii* replication.

Shortly after the invasion, *T. gondii* begins incorporating host cholesterol into the PVs, and additional radiolabeled cholesterol appears in various parts of the parasite, especially the plasma membrane and the rhoptries (9, 34). Our results showed that *T. gondii* cholesterol levels were significantly reduced in SREBP2 knockdown cells compared to WT cells, and the parasites exhibited morphological defects. This indicated that *T. gondii* required SREBP2 activation to acquire cholesterol.

Inhibiting host cholesterol transport with drugs reduced cholesterol on the host plasma membrane, which decreased *T. gondii* invasion (35). Similarly, we found that knocking down SREBP2 reduced membrane cholesterol accumulation and significantly decreased *T. gondii* invasion rates. Supplementing with additional cholesterol restored the invasion defect, which further confirmed the role of plasma membrane cholesterol in *T. gondii* invasion. Cholesterol on the plasma membrane was crucial for membrane stability, signal transduction, and pathogen invasion (36). In SREBP2 knockdown cells, plasma membrane cholesterol was significantly reduced which were useful for studying the role of membrane cholesterol in *T. gondii* invasion. These results suggested that upregulated SREBP2 facilitated *T. gondii* acquisition of cholesterol and highlighted the importance of further research about how cholesterol affects *T. gondii* invasion.

Our results showed that SREBP2 had no effect on the bradyzoites conversion rate, while it is crucial for bradyzoites' growth and replication. So inhibiting SREBP2 might limit the cyst development and further control the chronic toxoplasmosis. Previous studies reported that targeting cholesterol biosynthesis with HMGCR inhibitors (statins) in mice can treat brain cysts. These drugs were commonly used clinically to treat hypertension and hyperlipidemia, but their mechanism for treating brain cysts in mice remains unclear (18, 19). Our results indicated that regulating SREBP2 could affect cyst development. Further research about the role of cholesterol metabolism on *T. gondii* cyst development was warranted.

In summary, our study provided evidence that *T. gondii* acquired cholesterol by upregulating LDLR, HMGCR, SQLE, and DHCR7 through the activation of SREBP2. Targeting DHCR7 and SREBP2 exhibited antiparasitic effects. Our study uncovered a previously unrecognized role of SREBP2 in *T. gondii* infection and provided mechanistic insights into how SREBP2 inhibition exerted antiparasitic effects.

## Data availability

All data are contained within the article and/or its supplementary materials.

## Supplemental data

This article contains supplemental data.

## Conflict of interest

The authors declare that they have no conflicts of interest with the contents of this article.
